# Macrophage Extracellular Vesicles: Therapeutic Strategies for Corneal Fibrosis in Rare Diseases

**DOI:** 10.3390/biom16030346

**Published:** 2026-02-26

**Authors:** Haiming Li, Anne-Sophie Loewinger, Danial Roshandel, Yuan Fang, Jingjing You, Mark Daniell, Gink N. Yang

**Affiliations:** 1 Centre for Eye Research Australia, East Melbourne, VIC 3002, Australia; haiming.li@student.unimelb.edu.au (H.L.); alowinger@cera.org.au (A.-S.L.);; 2 Department of Surgery (Ophthalmology), University of Melbourne, Melbourne, VIC 3002, Australia; 3 Eye Clinic, Basel University Hospital, 4031 Basel, Switzerland; 4 Centre for Ophthalmology and Visual Science (Incorporating Lions Eye Institute), UWA Medical School, The University of Western Australia, Nedlands, WA 6009, Australia; 5 School of Medical Sciences, Faculty of Medicine and Health, University of Sydney Camperdown, Sydney, NSW 2050, Australia; 6 Lion’s Eye Donation Service Victoria, East Melbourne, VIC 3002, Australia; 7 Corneal Unit, Royal Victorian Eye and Ear Hospital, East Melbourne, VIC 3002, Australia

**Keywords:** corneal fibrosis, extracellular vesicles, Mac-EV, EV-based therapy, anti-fibrotic therapy, EV engineering

## Abstract

Corneal scarring (fibrosis) is a blinding condition affecting millions of sufferers worldwide. It is not only in common ocular injuries but also in genetically inherited rare diseases such as epidermolysis bullosa (EB), keratitis-ichthyosis-deafness (KID) syndrome and aniridia. In rare diseases like EB or KID syndrome, corneal fibrosis arises from chronic inflammation, structural instability and neuro-immune dysfunction driven by genetic mutations. Current therapies are not effective in addressing the needs of affected individuals due to limited efficacy nor the considerable side effects of treatment. Extracellular vesicles (EVs) from various cell types such as mesenchymal stem cells not only possess high biocompatibility but have shown promising results in limiting corneal fibrosis. Rather than targeting a single molecular signaling pathway, EVs which contain regulatory RNAs and proteins are hypothesized to target multiple pathways synergistically. Macrophage-derived EVs (Mac-EVs) with an immunomodulatory nature may offer a promising therapeutic effect for rare diseases. Various EV delivery platforms have been proposed in preclinical studies. However, not all of these delivery techniques are appropriate for the cornea in rare diseases. In this review, we delineate recent advances in understanding corneal fibrosis from a rare disease point of view, including the impact on corneal immune cells and nerves. We then provide critical considerations of therapeutic development for corneal fibrosis in rare diseases. Furthermore, we used this knowledge to comprehensively consider the various EVs, especially Mac-EVs, synthesis methods and delivery techniques. Ultimately, this review aims to enable biomolecule researchers to develop EV-based therapies that not only exert anti-fibrotic effects but also address clinical compatibility for corneal fibrosis in rare diseases.

## 1. Introduction

The cornea is a transparent tissue essential for vision. The cornea consists of five layers: epithelium, Bowman’s layer, stroma, Descemet’s membrane, and endothelium. Its highly ordered collagen structure and avascularity maintain transparency and refractive power [1]. Corneal clarity depends on an intact epithelial barrier, a specific corneal stromal deturgescence level, a stable extracellular matrix (ECM) and a controlled immune environment [2]. Any disruption to corneal stromal structure that leads to corneal opacity is considered clinically as corneal scarring. This process involves corneal fibrosis which occurs when injury-induced inflammatory signals activate transforming growth factor-β (TGF-β), platelet-derived growth factor (PDGF), and vascular endothelial growth factor (VEGF) or other fibrotic molecules, driving quiescent keratocytes to differentiate into fibroblasts and myofibroblasts and produce abnormal ECM, resulting in irreversible optical changes [3,4]. Corneal opacities represent the fourth leading cause of blindness worldwide, accounting for approximately 4% of all global blindness [5,6]. These processes occur in both common and rare forms of corneal fibrosis.

At the molecular and cellular levels, corneal fibrosis is closely linked to inflammatory response and neuroimmune crosstalk. An exposed stroma leads to the diffusion of soluble cytokines and growth factors between the epithelial and stroma, including TGF-β, PDGF, ILs, and other inflammatory mediators [7]. These signals act directly on stromal keratocytes while triggering an inflammatory response via the recruitment of immune cells [8,9,10]. The cornea relies on its natural immune privilege to maintain clarity, but injury or infection disrupts this state and triggers a rapid recruitment of innate and adaptive immune cells, including neutrophils, monocytes, macrophages, and T cells [11,12]. Excessive immune responses and prolonged keratocyte activation initiate the fibrotic cascade. In rare diseases, this process continues even in the absence of active injury or infection due to the underlying genetic defects, leading to recurrent or chronic fibrosis [13,14].

Most corneal scars are acquired due to diseases including infectious keratitis, trauma, surgery and chemical burns [15]. These conditions are usually triggered by injury or infection, or by chronic inflammatory states such as diabetes or neurotrophic keratitis. If infection and inflammation are controlled at an early stage, the epithelial basement membrane (EBM) has the potential to regenerate, which may allow partial regression of stromal opacity [8,16]. In contrast, corneal fibrosis from rare diseases arises from genetic abnormalities, such as corneal dystrophies, auto-immune related ocular surface diseases, or systemic genetic disorders affecting the cornea. Instability of basement membrane components, cell–cell junctions, or the cytoskeleton causes recurrent epithelial erosion and repeated EBM disruption, keeping TGF-β signaling pathway activated, leading to sustained fibrosis [17,18]. For these patients, current treatments mainly focus on supportive care. Artificial tears and lubricants improve the ocular surface, and corticosteroids can suppress inflammation and slow fibrotic progression to some extent [19,20,21,22]. However, these treatments are not a cure.

In the pursuit of safer and more accessible alternatives, especially for rare disease patients with chronic inflammation, extracellular vesicles (EVs) have shown promising results [15,23]. Mesenchymal stem cell-derived EVs (MSC-EVs) contain various anti-inflammatory factors and miRNAs that have the potential to restore the natural corneal immune privilege and to suppress myofibroblast formation [23]. EVs have low immunogenicity, can be engineered to increase delivery efficiency and be produced at a large scale, thus hypothesized to be suitable as drug carriers for ocular surface diseases [15]. Meanwhile, a variety of research on EVs in immune regulation and cell communication has provided a framework to understand the roles of EVs in inflammatory and fibrotic microenvironments [24].

In this review, we first delineate the role of immune response, macrophages and nerves in corneal fibrosis. Although these mechanisms are relevant to corneal fibrosis in general, this review focuses on rare corneal diseases, where fibrosis is driven by persistent genetic or developmental abnormalities. We then provide an overview of corneal fibrosis in rare diseases, including consideration for therapeutic development, current treatments and delivery platforms. Most importantly, we provide a comprehensive and critical summary of EVs, especially Mac-EVs as a potential therapy for corneal fibrosis.

## 2. The Role of Immune Response, Macrophages and Nerves in Corneal Fibrosis

### 2.1. Corneal Inflammation Leading to Fibrosis

Corneal fibrosis commonly begins with an epithelial injury, a basement membrane defect or impaired regeneration of EBM [8,25]. The cornea has a certain level of immune privilege at baseline, but once the epithelial barrier is disrupted, cytokines in the tear film and those released from damaged epithelial cells, such as IL-1, tumor necrosis factor-alpha (TNF-α), and TGF-β, can directly enter the exposed stroma and trigger a rapid inflammatory response [7,26]. Healthy EBM inhibits the diffusion of TGF-β and other fibrotic molecules, so EBM disruption prolongs the exposure of these signals in the stroma and becomes a key starting point for fibrosis. This mechanism is particularly important in rare diseases with structural protein defects, such as EB. Repeated epithelial breakdown leads to recurrent EBM disruption and sustained fibrotic signaling [27]. Keratocytes undergo rapid apoptosis after injury. Keratocytes and recruited bone marrow–derived immune cells are activated by inflammatory signals and shift toward fibroblast and myofibroblast phenotypes, gradually producing abnormal ECM [7,28,29]. These molecular mechanisms are largely shared between corneal fibrosis associated with rare diseases and fibrosis caused by common acquired conditions [17]. For example, in aniridia, limbal stem cell deficiency and epithelial instability lead to chronic inflammation and stromal scarring through pathways also seen in acquired corneal fibrosis [30].

The inflammatory response relies not only on cytokines but also on corneal immune cells. In vivo imaging studies showed that the cornea contained several immune cell populations under homeostasis, including dendritic cells for antigen presentation, tissue-resident macrophages, and patrolling T cells [11,31,32,33]. These cells rapidly accumulate at the injury site and release IL-6, IL-8, MCP-1, and complement components, which expand the inflammatory response. Persistent inflammation keeps NF-κB and related pathways activated, creating a long-lasting pro-inflammatory environment, extending keratocyte activation and ECM remodeling, resulting in fibrotic progression [4,34,35].

### 2.2. Involvement of Macrophages in Corneal Fibrosis

Macrophages are large, versatile immune cells that play a crucial role in the initiation, maintenance, and resolution of tissue injury [36]. Macrophages are hematopoietic cells derived from the mesoderm [37]. Tissue-resident corneal macrophages are established during embryonic development, mainly from the yolk sac or the fetal liver [38]. Corneal macrophage population can also derive from circulating blood monocytes that extravasate into the cornea via diapedesis in response to local environmental stimuli, subsequently differentiating into distinct subphenotypes [37,39]. Previous studies have linked about 50% of stromal corneal resident leukocytes to the monocyte/macrophage lineage [40], highlighting the important role of macrophages in corneal innate immunity.

In tissue repair and fibrosis caused by both common and rare diseases, macrophages obtain several key functions, including clearing debris by phagocytosis, tissue homeostasis, recruiting fibroblasts, and further stimulating keratocytes into myofibroblasts [41,42]. Hence, macrophages impact both the inflammatory phase and the subsequent phases of cell proliferation and ECM remodeling in corneal wound healing.

Macrophages have distinct phenotypes, each with different immunomodulatory properties [43,44]. M0 macrophages are considered the naïve, unpolarized subtype that serves as a baseline state and remains responsive to microenvironmental cues. Although considered quiescent, M0 macrophages secrete low levels of cytokines, including IL-5, IL-7, and IL-13, indicating a surveillance or homeostatic role and further suggesting these cells are not entirely inert [45]. Previous studies have also indicated that M0 macrophages actively contribute to tumor progression and immune modulation in the tumor microenvironment [46].

Classically activated M1 macrophages constitute the pro-inflammatory phenotype and are polarized by lipopolysaccharide (LPS) and interferon-gamma (IFN-γ) [47]. By releasing pro-inflammatory cytokines including IL-1β, IL-6, IL-12, IL-23, TNF-α, and inducible nitric oxide synthase (iNOS), M1 macrophages facilitate the removal of pathogens at the wound site [45,48,49,50].

IL-4, IL-10, or IL-13 induce alternatively activated M2 macrophages, representing the anti-inflammatory phenotype [48]. M2 Macrophages are associated with tissue repair processes via ECM remodeling and release a subset of anti-inflammatory cytokines such as IL-10, CCL18, and TGF-β [48,51]. M2 macrophages can be further divided into subphenotypes including M2a, M2b, M2c, and M2d [52,53]. M2a macrophages are induced by IL-4, while IL-10 polarizes M2c macrophages [54]. The other M2 macrophage subsets, M2b, and M2d, have also been reported, but the induction of these cells appears to be much more complex and remains to be further studied [52,55].

Once polarized to M1 or M2 states, macrophages exhibit phenotypic plasticity and are capable of depolarizing into M0 and subsequently cycling back to M1 or M2 depending on the cytokines presented within the tissue environment [56].

### 2.3. Involvement of Corneal Nerves in Corneal Fibrosis

The cornea is the most densely innervated tissue in the human body [57]. Nerve fibers are spread out throughout the posterior corneal epithelium and anterior stroma but are absent in the endothelium [58,59]. Corneal nerves provide sensation and communication with immune cells through neuropeptides such as calcitonin gene-related peptide (CGRP) and substance P (SP) to maintain immune balance on the ocular surface [60].

Upon inflammation, corneal nerves release SP, which in synergism with epidermal growth factor (EGF) stimulates epithelial proliferation and wound healing [61,62]. Furthermore, SP has been shown to induce keratocyte contraction, stimulate the secretion of collagen types I, II, and V, and upregulate alpha-smooth muscle actin (α-SMA) and fibronectin, thereby promoting a shift in stromal keratocytes towards a pro-fibrotic, myofibroblast phenotype, ultimately contributing to corneal fibrosis [63]. Other neuromediators released by corneal nerve fibers include neurokinin A, CGRP, and nerve growth factor (NGF), which have also been shown to provide trophic support and stimulate wound healing [64,65,66].

Rare ocular surface disorders such as EB, particularly when chronic, facilitate corneal nerve damage by inducing persistent epithelial disruption and continuous (subtle) inflammation without active injury or infection [60]. Consequently, this process perpetuates a cycle of injury and abnormal repair, which may lead to fibrosis [62,67].

## 3. Corneal Fibrosis in Rare Diseases

### 3.1. Rare Diseases and Epidermolysis Bullosa (EB)

Instead of acquired injury or infection, corneal fibrosis from rare diseases often arises from chronic autoimmune response and defected structural proteins due to genetic abnormalities. EB, with an overall estimated incidence of approximately 19.57 and 11.07 per 1 million live births and per 1 million population in the United States [68], stands out as a significant corneal fibrosis causing rare disease on the NCBI rare disease database [17], with frequent corneal involvement and a relapsing, chronic, and progressive course [22].

In junctional EB (JEB), mutations in *LAMA3*, *LAMB3*, or *LAMC2* cause loss or dysfunction of laminin-332 [27,69,70]. Laminin-332 is an essential part of the corneal epithelial adhesion complex, and its deficiency weakens the attachment between the epithelium and the EBM, making the epithelium extremely sensitive to minor mechanical friction [25,71]. Clinical signs include recurrent erosions, persistent epithelial defects, and chronic inflammation [71,72,73]. With repeated injury, the basement membrane becomes chronically disrupted, prolonging the exposure of TGF-β in the stroma and gradually pushing the cornea into a fibrotic process.

In dystrophic EB (DEB), mutations in *COL7A1* lead to defects in type VII collagen [74]. Collagen VII forms anchoring fibrils and is critical for the stability of the EBM-anterior stroma [74]. When collagen VII is absent, anchoring fibrils are severely reduced, leading to unstable epithelial–stromal adhesion and recurrent subepithelial separation. This epithelial fragility predisposes the ocular surface to repeated erosions and chronic inflammation persists [75,76].

Apart from EB, other rare diseases such as aniridia caused by PAX6 mutation (1 in 40,000 to 100,000 individuals) [77,78,79], KID syndrome (less than 1 in 1,000,000 individuals) [80,81], and hereditary sensory and autonomic neuropathy IV (HSAN-IV, 1 in 600,00 to 950,000) [82,83] also have the potential to cause corneal fibrosis. These conditions often involve defects in epithelial barrier function, abnormal nerves, impaired immune regulation, or dysfunction of limbal epithelial stem cells, leaving the cornea in a chronically fragile microenvironment with repeated cycles of inflammation and repair. Even with adequate lubrication, anti-inflammatory treatment, and infection control, the EBM is unable to regenerate, wounds fail to close properly, and inflammation and fibrosis form a self-sustaining cycle. These conditions require treatments that can compensate for structural defects, stabilize the basement membrane, reduce chronic inflammation, and regulate fibrosis. Gene therapy has shown potential as a treatment for rare diseases, but it is limited by high cost and complex procedures [84,85].

### 3.2. Considerations of Therapeutic Development for Rare Diseases

**Figure 1:**
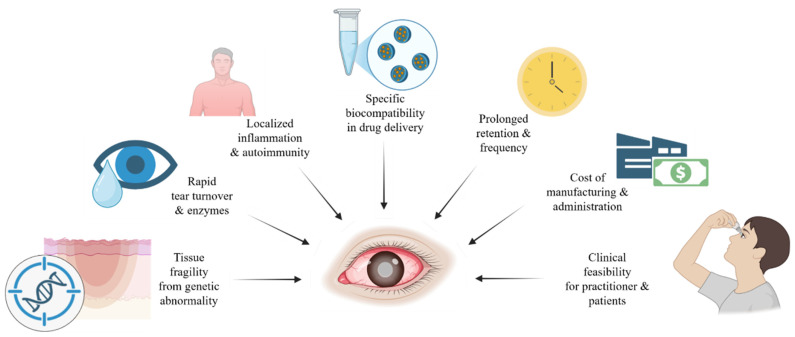
Specific considerations of therapeutic development for the cornea in rare diseases. Patients with corneal wounds in rare diseases require eye care that is fit for a multitude of purposes. These include the consideration of tissue fragility from genetic abnormality, rapid tear turnover and enzymatic activity, localized inflammation and autoimmunity, specific biocompatibility in drug delivery, prolonged drug retention time, high cost of drug manufacturing, and feasibility for clinicians and patients.

Rare diseases that lead to corneal fibrosis pose unique challenges for therapeutic development. The ocular surface is a dynamic environment. Topical formulations are exposed to rapid tear turnover, blinking, and enzymatic activity, which can dilute or remove drugs within minutes [86]. To overcome this, delivery systems are designed to have an extended retention time through mucoadhesive polymers, in situ gelling formulations, nanoparticle carriers, or biologic scaffolds. Each system requires careful optimization of viscosity, osmolarity, and pH to avoid causing further irritation or mechanical stress to an already compromised epithelium [86,87]. Together, these challenges highlight the barriers to therapeutic development for corneal fibrosis in rare diseases, which are summarized in Figure 1.

Another difficulty arises from the fragility and heterogeneity of the disease itself. For instance, in DEB, loss of type VII collagen creates extreme epithelial instability and unpredictable wound patterns [72]. Drug penetration, bioavailability, and potency can vary significantly depending on epithelial integrity, tight-junction health, and local inflammation [88].

It is estimated that 80% of rare diseases are caused by genetic mutations. Gene-based therapies add further complexity as viral and non-viral vectors must be engineered to transduce corneal cells efficiently without provoking excessive inflammation or off-target effects. In rare diseases caused by loss of structural proteins, gene therapy could theoretically correct the defect, but current approaches are costly, complex, and mostly in early development. Achieving sufficient expression while maintaining safety remains a major translational barrier [89]. Gene therapy is also difficult to apply in families already facing heavy financial burdens [90]. From the initial concept to clinical application, the development of a gene therapy can take decades [91]. A large proportion of the cost and time is consumed during the late-stage clinical trials. The treatment itself may cost up to one million USD per administration [91]. Recently, the European Medicines Agency (EMA) and the U.S. Food and Drug Administration (FDA) approved Vyjuvek as a topical gene therapy for EB, with an official wholesale price of $24,250 per vial [92]. However, due to the protein deficiency, patients are likely to require lifelong treatment. According to a study published in *JAMA Dermatology*, the estimated lifetime cost of therapy may reach $15 to $22 million, which is more expensive than any one-time gene therapy for other diseases [93].

Manufacturing logistics can also limit progress. Many rare-disease approaches rely on autologous or small-batch production that require rigorous GMP compliance. Given the small patient numbers, the individual cost and manufacturing burden are disproportionately high, and batch-to-batch consistency is difficult to achieve [94].

Building relevant and useful preclinical models is another major barrier. Conventional animal models rarely mimic the chronic fragility, blistering, non-healing wounds, or fibrosis typical of DEB or severe ocular surface failure. Ex vivo human corneas, patient-derived organoids, or advanced biomimetic platforms are powerful but technically demanding, and require careful validation to predict toxicity, penetration, and efficacy [95]. Finally, clinical trial design must adapt to the reality of small cohorts with widely variable baseline severity. Sensitive imaging and molecular biomarkers, measurement tools, and patient-specific functional endpoints often need to be validated through natural history studies [96].

### 3.3. Current Ocular Treatments and Drug Delivery Options for Rare Diseases

For corneal rare disease patients represented by EB, current treatments are still limited on supportive methods, with the main goals to reduce mechanical friction, stabilize the ocular surface, and suppress inflammation and secondary infection as much as possible [22].

The most common treatments are lubricants, including preservative-free artificial tears, gels, or ointments. These products can temporarily improve tear-film stability and reduce epithelial friction [22,97]. However, the duration is short and frequent use is needed. For EB patients with fragile periocular skin and eyelids, high dosing frequency is a burden. Antibiotic eye drops are essential when treating repeated epithelial defects and can reduce the risk of infection caused by epithelial gaps. Topical corticosteroids are also used to suppress inflammation and slow fibrosis, but long-term use may cause raised intraocular pressure, so clinical monitoring is crucial [98]. Non-steroidal anti-inflammatory drugs (NSAID) are sometimes used for corneal pain, but the anti-inflammatory effect is limited, and the irritation needs careful assessment [99].

An eye drop made from human amniotic membrane (AM), which contains laminin-332 and has anti-inflammatory and anti-fibrotic effects, has relieved ocular manifestation in a child with JEB [100]. AM extract has natural repair potential and shows epithelial-healing effects in dry eye and alkali burns, but contains multiple growth factors such as EGF, FGF, and TGF-β [101]. Some factors may have double-edged effects under chronic inflammation or EBM defects, so individual evaluation is needed. Overall, these treatments help relieve symptoms and reduce acute injury but cannot correct epithelial instability caused by structural-protein defects and cannot stop the fibrosis caused by long-term repeated damage.

There are also surgical approaches for rare diseases. Corneal transplantation in keratoconus (approximately 1 in 2000 individuals) shows high survival rate (89% at 10 years and 49% at 20 years) [102]. However, in other rare diseases, surgical approaches outcomes remain limited. Autologous cultivated limbal epithelial cells transplantation has shown surface restoration in isolated cases [103]. In contrast, penetrating keratoplasty (PK) and keratoprosthesis transplantation in children with EB carry high risks, including corneal melt, graft failure, and phthisis bulbi, as reported in multiple case studies [104,105,106]. For rare disease patients, general anesthesia itself is a significant risk, as they may have lower surgical tolerance. For EB patients, there is a risk of blister formation during intubation [81]. Therefore, the development of non-invasive therapeutic strategies is particularly important for this patient population.

### 3.4. Drug Delivery Options for Rare Diseases

Due to the fragile ocular structures of rare disease patients, efficient delivery methods are particularly important. New carrier forms can help combine multiple drugs, providing both anti-inflammatory and anti-infective effects [107]. In recent years, advanced drug delivery systems including hydrogels and nanoparticles have been applied to the eyes. These systems can increase drug adhesion, extend retention time, or provide sustained release, reducing the daily treatment burden. More importantly, these materials have the potential of combining different drugs to form composite carriers with anti-inflammatory, anti-infective, and epithelial-healing functions [107].

Hydrogel materials are known to possess high biocompatibility due to the high-water content and the ability to form a soft protective layer on the corneal surface [108]. Hydrogels also have the potential to improve tear-film stability and act as slow-release carriers to extend drug retention. For example, a hydrogel made of the anionic polysaccharide gellan was shown to reduce corneal scarring in a microbial keratitis model when used with corticosteroids and antibiotics [109].

Nanoparticles and lipid-based nanoplatforms have also been proposed as a drug carrier for rare diseases due to its highly tunable structure, which can improve the solubility of hydrophobic drugs, increase corneal penetration, and achieve a more controlled release profile. Nanocarriers with adjustable pore size and surface area, such as mesoporous silica, can increase the effective surface area, allow co-loading of multiple molecules, and improved binding to bacterial toxins or inflammatory factors, providing more efficient local control in corneal infection and fibrosis [110]. Cationic nanostructured lipid carriers (CNLC) have shown superior efficacy due to the electrostatic bio-adhesiveness to ocular tissues, thereby enhancing drug delivery [111]. Lipid-based nanoparticles (LNPs) are promising carriers for ocular drug delivery, as LNPs can enhance drug solubility, improve bioavailability, and provide sustained release. Nanogels have the ability to encapsulate drugs within a complex three-dimensional cross-linked polymer network, providing a platform for controlled and sustained drug release, ultimately improving the bioavailability of ocular medications [112]. Wang et al. have developed a novel dexamethasone-loaded, ROS-responsive, controlled-release nanogel, called DEX@INHANGs to target corneal neovascularization [113]. DEX@INHANGs inhibited the formation of corneal neovascularization with lower cytotoxicity to corneal cells and good biocompatibility compared with free dexamethasone, demonstrating its potential as an ocular delivery system [113].

In addition, cell-derived nanocarriers have recently shown therapeutic potential due to their natural biocompatibility and multifunctional signaling capacity. These biological vesicles are discussed in detail in the following section [15,114,115]. Overall, current supportive treatments and emerging delivery platforms for corneal rare diseases are summarized in Table 1.

## 4. Extracellular Vesicles for Corneal Fibrosis

### 4.1. Involvement of EVs in Corneal Wound Healing

EVs are categorized into three subtypes: exosomes, microvesicles, and apoptotic bodies. Exosomes, ranging from 30 to 150 nm in diameter, originate from the inward budding of endosomal membranes and are secreted upon fusion of multivesicular bodies (MVBs) with the plasma membrane. Microvesicles (100–1000 nm) are shed directly from the plasma membrane through outward budding. Apoptotic bodies, released during the late stages of programmed cell death, are highly heterogeneous in size (50–5000 nm) [24].

EVs are encapsulated by a single lipid bilayer and contain a diverse cargo of proteins, nucleic acids, and lipids [24]. These molecules are delivered to recipient cells and mediate various forms of intercellular communication. Among the different EV subtypes, exosomes have attracted increasing attention in precision medicine due to their small size, membrane stability, and enriched bioactive content [121].

Corneal fibrosis is an inflammation-mediated and multi-pathway regulated process, and it involves a series of interreacted pathways. Because of its complexity, a single-target small molecule or protein drug is unable to address all the fibrotic signals at the same time. Therapies that only block TGF-β are not able to fully stop fibrosis. EVs are natural carriers containing microRNAs (miRNA), proteins, and membrane components, which can act on multiple pathways [24,121,122]. EVs are also natural communication tools in the cornea. In the normal state, EVs participate in material exchange and signal transfer between the tear film, epithelium, and stroma [121]. In disease conditions such as dry eye, infection, keratoconus, and glaucoma, the composition of EVs undergo pathological change and contributes to inflammatory and fibrotic signals [121].

Early animal studies found vesicle-like structures at the EBM of healing corneas, suggesting a possible role of EVs in wound repair [123,124]. EVs from corneal myofibroblasts promoted cell proliferation, migration, and movement in epithelial cells model [125]. EVs from epithelial cells also influenced myofibroblast differentiation [126]. These findings suggest that EV-mediated communication between the epithelium and stroma affects wound healing and scar formation. EVs in the cornea may also bind ECM components or express surface proteases that modify ECM proteins and take part in cell–ECM interaction [121]. These findings indicate that EVs are important communicators in the cornea, and targeted modification of EVs may allow more precise control of the fibrotic process.

Compared with free proteins or small molecules, EVs have been shown to remain stable on the ocular surface, diffuse more easily into the stroma and be taken up by local cells [24]. EV shows better biological safety than synthetic nanoparticles [24].

### 4.2. Specific EVs for Corneal Fibrosis

**Figure 2:**
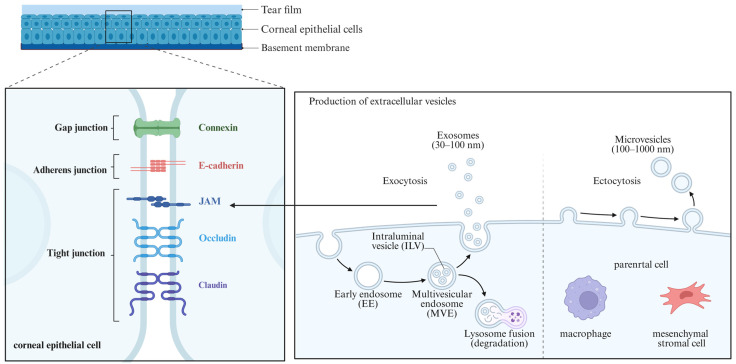
Consideration of types and sizes of EVs for treating corneal fibrosis.

From a biological perspective, all EVs are lipid-bilayer vesicles secreted by cells, but not all types are practical for corneal fibrosis treatment. Most ophthalmic and corneal studies mainly use the small-EV fraction enriched in exosomes, with a diameter around 50–150 nm, rather than large microvesicles or apoptotic bodies [115]. The biological and anatomical considerations underlying the preferential use of small EVs in corneal fibrosis studies are illustrated in Figure 2.

First of all, exosomes resemble natural signaling particles used for cell–cell communication. Exosomes are generated through the endosomal–multivesicular pathway and contain specific membrane proteins and miRNAs, which reflect programmed secretion patterns during stress, inflammation, or repair in the parent cell [24]. In 1987, Zieske et al. described the presence of membrane-bound particles in the corneal stroma three days after keratectomy, providing one of the earliest pieces of evidence for the involvement of EVs in corneal wound healing [124]. In current studies, whether the EVs come from MSCs, CSSCs, or immune cells, most vesicles that are isolated, characterized, and applied in animal corneal models belong to this small vesicle group of around 100 nm [23,115,127]. This EV type is more controllable with standard production, and more suitable for engineering and drug loading.

Secondly, based on corneal anatomy and delivery routes, small-size EVs distribute more evenly across corneal layers and are taken up more easily by key cells. The corneal epithelial cells with tightly bound adhesion proteins act as a major barrier to drug absorption [128]. The permeability of the cornea is very limited, approximately one-tenth that of the sclera and one-twentieth that of the conjunctiva [129]. However, EVs are more likely to cross ocular surface barriers than ionic or hydrophilic compounds due to the phospholipid bilayer membrane structure [130]. However, the ability of EVs to penetrate the ocular surface barrier remains limited. Transmission electron microscopy has shown that EVs are unable to pass through the EBM [131]. In rare diseases with severe EBM disruption, EV delivery may be more effective. EVs can also indirectly reduce stromal fibrosis by modulating epithelial inflammation [115,132]. EV size also plays an important role [133]. Smaller EVs are more easily to be taken up by recipient cells while larger EV subpopulations failed to exert functional effects [133]. Thereby, optimizing EV purification and selecting the appropriate size range is critical for maximizing delivery efficiency and therapeutic efficacy [134].

Mesenchymal stromal cells and macrophages are illustrated as parental cells of EVs. Current corneal studies mainly utilize exosome-enriched small EVs (50–150 nm) due to more mature purification workflows and stable cargo composition. Tight junctions in corneal epithelium forms an initial barrier, while epithelial or EBM disruption permits deeper stromal access and cellular uptake of EVs.

EVs from different cell types also show functional differences. EVs show anti-inflammatory, pro-epithelial repair, and anti-fibrotic effects in several corneal disease models. MSC-EVs reduce inflammatory cytokines, limit immune-cell infiltration, promote M2 macrophage polarization, shift inflammation toward repair, and reduce scar formation [15]. These early anti-inflammatory and immune-modulating effects prevent uncontrolled repair and support non-scarring regeneration.

The early focus of CSSCs research was to produce a cell-based therapy [114]. CSSCs are similar to stromal progenitor cells, which can differentiate into keratocytes and secrete ECM components of the corneal stroma [114]. Later studies found that the anti-fibrotic and immune-modulating effects of CSSCs are closely related to the exosomes secreted by CSSCs. Study also showed that blocking miRNA packaging and exosome release reduced the scar-suppressing ability of CSSCs, suggesting that CSSC-EVs contribute to the therapeutic activity of CSSCs [135]. Further studies identified anti-fibrotic molecules such as miR-29a and miR-381, which suppressed the inflammatory phenotype of macrophages, reduced fibrotic markers in stromal cells, and decreased scar formation in animal models [136]. Although CSSC-EVs show therapeutic potential, the large-scale expansion system and the downstream preparation and characterization of EVs still lack mature industrial standards compared with MSCs. Yam et al. recently proposed a GMP protocol for the isolation, expansion, and cryopreservation of CSSCs, and also developed in vitro quality control indicators that reflect the anti-scarring potency of CSSCs in vivo, providing a basis for future standardized upstream and downstream processes [114].

Although the current focus of CSSC research is still to produce a cell-based therapy, patients with rare diseases who have unstable corneal structure, such as EB, often cannot tolerate repeated stromal injection. Cell transplantation also faces attachment, survival, and handling risks. Therefore, CSSC-based exosome therapy may be a better alternative for rare-disease patients.

In current EV studies related to corneal fibrosis, macrophages are mainly used as responders to treatment [137]. Researchers usually assess the polarization of macrophages toward M1 or M2 phenotypes to evaluate whether the local immune microenvironment moves from an inflammatory state to a repair state [23]. Therefore, from a mechanistic point of view, directly using Mac-EVs as a therapeutic tool is also worth exploring.

### 4.3. Current Developmental Progress of EVs for Corneal Fibrosis

Current EV studies related to corneal fibrosis mainly focus on three sources: MSC exosomes, CSSC exosomes, and Mac-EVs. Allogeneic EVs offers advantages in accessibility, standardized production, and flexible donor selection [15]. The risk of immune rejection remains a concern, but the immune-privileged nature of the cornea may reduce this drawback. In contrast, autologous EVs provide better compatibility but remain less explored due to challenges in obtaining patient-derived cells. A study involving the intrastromal injection of autologous adipose-derived MSCs reported no inflammatory response and moderate visual improvement, supporting the therapeutic potential of autologous EVs [138]. Key EV sources, experimental models, and the associated anti-fibrotic mechanisms reported in corneal studies are summarized in Table 2. MSC-EVs are the most well-studied and evidence-supported approach, and the anti-inflammatory, pro-repair, and anti-fibrotic effects have been repeatedly validated both in in vivo and in vitro models [15]. MSC-EVs suppress pro-inflammatory cytokines and reduce the expression of α-SMA, Col3, and FN. These effects are mainly mediated by miRNAs such as miR-21, miR-23a, and miR-29, as well as other specific protein factors [15].

Bonelli et al. created a 1-heptanol-induced epithelial injury model in ex vivo human corneas. Faster epithelial cell migration from in vitro scratch assays and quicker epithelial wound closure were observed in the ex vivo corneas and when MSC-EVs (~80 nm) were applied [140]. Tang et al. showed that iPSC-MSC exosomes promoted proliferation and migration of corneal epithelial cells and stromal stem cells in vitro, and reduced fibrosis and neovascularization while improving corneal clarity in a rat anterior stromal injury model, mainly by miR-432-5p [139].

In addition to common MSC markers such as CD73, CD90, CD105, and CD140b/PDGFRβ, Pax6 and ABCG2 are key markers for CSSC [143]. CSSCs are mainly located in the anterior stroma near the epithelial limbus, and this specific niche allows CSSCs to respond quickly to corneal injury [144]. Exosomes from CSSCs extracted from donated human corneas promoted human corneal epithelial wound healing in vitro [145]. When miRNAs were silenced by siRNA, the anti-fibrotic effect of CSSC-EVs was reduced in a mouse corneal injury model, indicating that the effect depended on miRNAs within the EVs [115]. Further characterization of these EVs suggested the involvement of miR-29a and miR-381-5p [136].

In current corneal fibrosis studies, macrophages are often used as indicators of immune microenvironment changes after treatment rather than direct therapeutic sources. However, in fibrosis studies of other tissues, such as the peritoneal fibrosis model, Mac-EVs showed an anti-fibrotic effect mediated by miR-204-5p through FOXC1 inhibition [141], and FOXC1 also promotes fibrosis in the cornea [146]. M2-EVs were tested in in vitro models of human corneal endothelial inflammation and wound healing [142]. In LPS-stimulated and scratch-wounded human corneal endothelial cells (hCEnCs), M2a-exo and EGF-preconditioned M2a-exo reduced inflammatory marker expression and restored cell proliferation [142]. These findings suggest that Mac-EVs have may have a therapeutic potential for corneal fibrosis.

### 4.4. Potential Delivery Methods of EVs for Corneal Fibrosis

EVs can be delivered to cornea through three main approaches, including topical application, hydrogel-based delivery, and subconjunctival injection (Table 3). Topical application represents the most common and least invasive approach [147]. For instance, topical lyophilized canine MSC-EVs applied once daily for one week in a rabbit stromal ulcer–induced corneal fibrosis model markedly accelerated re-epithelialization and preserved a homogeneous stromal architecture by day 5, whereas saline controls showed persistent epithelial defects with full-thickness hyperreflective stromal fibrosis [148]. Nevertheless, the effectiveness of eye drops remain constrained by rapid precorneal clearance and the cornea barriers, resulting in poor ocular bioavailability of <5% [149].

A limitation of EV-based therapy is the need for repeated administrations, as EVs tend to be rapidly cleared from the body when applied alone [155]. In ocular delivery, EVs may be quickly washed away from the ocular surface due to frequent tear turnover. However, in vitro studies have shown that incorporating EVs into hydrogels can significantly prolong their retention and release time on the ocular surface, extending it up to ten days [156]. miR-24-3p–rich adipose-derived mesenchymal stem cell-derived exosomes delivered via a hyaluronic acid-based hydrogel demonstrated sustained uptake by rabbit corneal epithelial cells for at least 7 days in vitro [151]. In a rabbit alkali-burn model, it suppressed stromal fibrosis as indicated by decreased α-SMA expression compared with PBS controls. Injectable delivery methods, including subconjunctival injections, have also been used to deliver EVs directly to periocular tissues. Although topical dosing (days 0, 1, and 3) similarly produced anti-fibrotic effects, only subconjunctival delivery induced a regenerative M2 macrophage response (Arg-1), potentially due to deeper stromal penetration, although the mechanism was not investigated in the study [157]. Despite its efficacy, subconjunctival administration is more invasive and carries a higher risk of procedure-related injury [158].

## 5. Mac-EVs and Exosomes

### 5.1. Types of Mac-EVs and Exosomes

Mac-EVs, including exosomes, are progressively gaining significance as cellular waste products to crucial mediators that exert immunomodulatory functions [159,160,161,162]. Macrophages generate a significant proportion of circulating microvesicles under both physiological and pathological conditions. Mac-EV cargo includes exosomes and other EVs that reflect the highly diverse macrophage activation states, functional specialization, and the dynamic cell cycle [163,164]. Macrophage-derived exosomes (MDE) encapsulate distinct bioactive molecules, including lipids, miRNAs, circular RNAs (cirRNAs), and proteins [165,166]. Delivery of MDE from producer to effector cells facilitate intracellular communication and thereby contribute to various biological processes [165,166,167,168].

Macrophage polarization is influenced by different cell cycle phases, with G1-phase cells tending toward pro-inflammatory responses and S/G2/M-phase cells displaying more anti-inflammatory features [164]. Treatment of macrophages with LPS or IFN-γ results in an accumulation of p21 mediated by the release of reactive oxygen species, ultimately facilitating cell cycle arrest and polarization to the pro-inflammatory M1 phenotype [169,170]. Meanwhile, IL-4 can promote S-G2/M-biased gene programs linked to tissue remodeling in macrophages as well as shift the cell cycle phase towards the G2/M phase [164]. Interestingly, single-cell approaches have demonstrated a remarkable heterogeneity of cell cycle-dependent macrophage responses [164]. Reflecting on this cell-cycle-specific variety of Mac-EV cargo, we hypothesize that Mac-EVs are particularly well-suited to target rare corneal fibrotic diseases. We postulate that by tightly shaping cell-cycle dependent cues an active shift toward tissue-remodeling could be accomplished. Specifically, IL-4–driven S/G2/M-biased Mac-EVs are enriched in anti-inflammatory and regenerative factors, offering a potential strategy to modulate excessive immune responses and aberrant tissue remodeling that underlie corneal fibrosis in rare diseases. Although numerous studies have characterized Mac-EVs across various subphenotypes, specific Mac-EV markers that highlight the individual activation and functional specialization state remain to be identified in future research [52,53,54].

**Figure 3:**
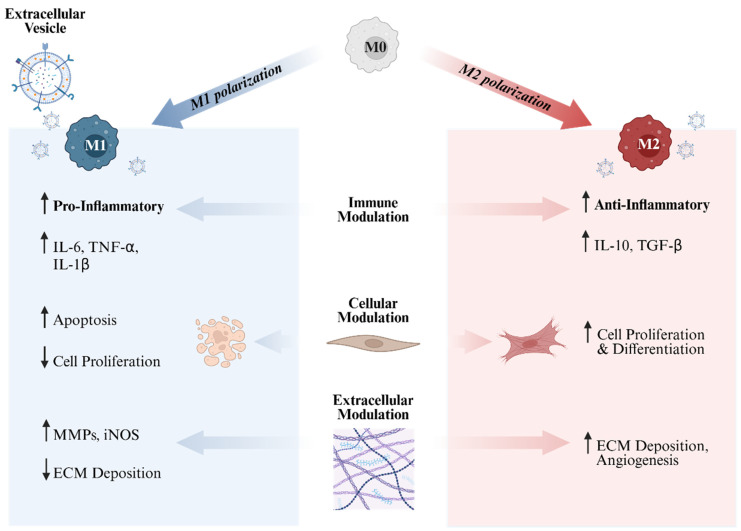
Macrophage-derived extracellular vesicles (Mac-EVs). M1 macrophages facilitate inflammation by secreting pro-inflammatory cytokines, promoting apoptosis, and inhibiting tissue remodeling. M2 macrophages support tissue regeneration by secreting anti-inflammatory cytokines, stimulating cellular proliferation, and promoting ECM depositions.

Mac-EVs have been implicated in the pathogenesis of fibrosis across multiple organ systems, including the lungs, kidneys, heart, connective tissue, and the vascular system [171,172,173,174,175]. Mac-EVs are known to target various pathways, including Smad7, Smad3, Wnt/β-catenin, and TGF-β [171,172,173,174,175]. The functional impact of Mac-EVs on fibrosis is closely associated with macrophage polarization (Figure 3). In in vivo and in vitro models of peritoneal fibrosis, exosomes from macrophages carried miR-204-5p and regulated FOXC1-related pathways, leading to reduced collagen deposition and reduced fibrotic markers [141]. Kim et al. suggested a modulatory role of MDEs in cutaneous inflammation and angiogenesis by delivering cytokines, including IL-4, CXCL12, and basic fibroblast growth factor (bFGF) [176]. This led to the conversion of M1 macrophages to M2 macrophages directly at the wound site, thereby facilitating an anti-inflammatory immune response and promoting wound closure [176].

Macrophages are major participants in corneal innate immunity, and the recruitment and activation of macrophages are key steps in wound closure and stromal remodeling [177]. Lan et al. found that a forkhead domain inhibitor reduced fibrosis in a rat alkali-burn model, suggesting that Mac-EVs may also have potential in corneal fibrosis [178]. In a corneal endothelial injury study, exosomes from M2 macrophages reduced inflammation in corneal endothelial cells and promoted survival and proliferation [142].

In addition, the engineering of Mac-EVs has also been widely reported. Mac-EVs can be engineered by enriching specific miRNA or proteins, or by attaching targeting ligands to the surface. These modifications have been shown to enhance EV accumulation in specific tissues [179,180,181]. Engineering macrophage derivatives with IL4RPep-1 peptide, which binds to IL-4 receptor (IL4R), were shown to enhance the targeting ability of Mac-EVs toward tumor tissues [182]. In osteoarthritis, specific surface engineering of Mac-EVs further increased the efficacy of drug delivery in the subchondral bone [183].

### 5.2. Synthesis and Characterization of EVs

The separation and characterization of Mac-EVs and exosomes require careful consideration of both biological and methodological variables to ensure reproducibility and functional relevance. Defining the macrophage source (i.e., primary cells versus cell lines such as THP-1) as well as the differentiation and polarization protocols is essential. Furthermore, it is important to consider that variability in the primary Mac-EV-containing matrix (e.g., cell culture medium, blood, tissue), the Mac-EV separation method, and Mac-EV storage [184]. These variables can influence Mac-EV production and/or composition, underscoring the importance of comprehensive documentation for traceability and repeatability [184,185].

Although various methodologies for separating and analyzing Mac-EVs have been described in the literature, standardization of these methods remains warranted [184]. To ensure the optimal enrichment of Mac-EVs, separation protocols should be selected based on the intended downstream application [186]. Ultracentrifugation remains the predominant method employed for EV separation [187,188]. However, it may also result in co-isolation of other non-exosomal components, including proteins and cellular debris [187,189,190]. Density gradient centrifugation, filtration, size-exclusion chromatography (SEC), and sequential or combined methodologies are also frequently used for Mac-EV separation [187,191].

For further downstream applications, it is essential to characterize the separate Mac-EV populations. The latest edition of the Minimal Information for Studies of Extracellular Vesicles provides an extensive guideline for EV characterization, highlighting the assessment of morphology and particle size, and the quantification of lipids, proteins, and RNAs as key markers. Commonly used morphological characterization techniques include scanning electron microscopy, transmission electron microscopy, and cryo-electron microscopy [189,192,193]. Identification of protein markers is usually performed using Western blotting or enzyme-linked immunosorbent assays (ELISA) [194].

### 5.3. Potential Delivery Methods of Mac-EVs

Optimal delivery of Mac-EV requires consideration at nanoscale dimensions, membrane chemistry, and clearance dynamics, as these properties collectively influence EVs-cell interaction and tissue penetration. Small EVs (<200 nm) diffuse well when delivered as eye drops, but possess low bioavailability of the contents due to rapid clearance from the ocular surface [195]. Hydrogel-based delivery systems, including hydrogel contact lenses, can help prolong Mac-EV and enable sustained release, thereby enhancing delivery to the corneal stroma [196,197]. In contrast, large EVs (>200 nm), such as microvesicles, carry substantial cargo but may exhibit poor tissue permeability [198]. Large EVs are unable to penetrate deeply into stroma due to the densely packed ECM, restricting access to fibrotic cells [199]. Consequently, barrier-bypassing routes such as subconjunctival injections are generally more suitable for this EV population; however, not appropriate for rare diseases. EV surface properties further modulate delivery efficiency. For example, EVs with highly glycosylated surface proteins may become entrapped within the mucin-rich tear film, reducing diffusion ability and limiting penetration when delivered as eye drops [200,201]. The short half-life of EVs, typically approximately 2–20 min, can also limit the retention following topical administration and may necessitate repeated dosing [202].

### 5.4. Targeting EB-Associated Rare Diseases Using Mac-EVs

Unlike gene therapy, which is often developed for specific genetic mutations and requires personalized production, EV-based therapy may offer a more generalized approach. EVs participate in multiple phases of wound healing, including promoting hemostasis, modulating inflammation, and inducing cellular proliferation and tissue remodeling [203]. Studies have shown that EVs can reduce organ fibrosis in various preclinical models by inhibiting myofibroblast differentiation and degrading the ECM [204]. Moreover, EV production benefits from established purification and engineering protocols that support scalable manufacturing. Shared production platforms across different EV-based products allow cost amortization, significantly reducing the final price [205]. Compared to gene therapy, which can cost millions of dollars per patient, current clinical-grade EVs such as MSC-EVs are already available on the market at a much lower cost, often priced in the range of a few hundred Euros per vial [205].

Previous studies have linked Mac-EVs and MDEs to several key clinical features of EB as a model disease for corneal scarring in rare fibrotic diseases. For instance, Mac-EVs and MDEs have been reported to improve wound healing by reducing TNF-alpha and IL-6 expression and increasing miR-222-3p expression [206,207]. Mac-EVs have also been shown to induce macrophage phenotype switching from M1-like to M2-like in a murine model, thereby enhancing angiogenesis, re-epithelialization, and collagen deposition, ultimately facilitating wound healing [176,207]. Complementing these findings, Zhou et al. demonstrated that keratinocyte-derived exosomes can reprogram wound-edge macrophages to resolve inflammation and promote tissue regeneration, supporting the feasibility of EV-mediated immune modulation within skin wounds [208]. Given the clinical features of EB, including recurrent surface defects and chronic inflammation, EV-mediated modulation could offer a promising therapeutic approach for directly targeting functional wound repair.

Furthermore, bone marrow-mesenchymal stem cell-derived (BM-MSC) EVs have been shown to transport C7 protein and mRNA in vitro, stimulating fibroblasts to produce new C7 in patients with recessive dystrophic EB (RDEB) [209]. Given the additional immunomodulatory potential, Mac-EVs could therefore not only accelerate wound healing but also address the root of EB by delivering targeted molecular cargo and thus remain to be further investigated.

## 6. Conclusions

Corneal fibrosis from rare diseases such as EB represent an unmet clinical need at the intersection of genetics, inflammation, and tissue remodeling. Although some topical therapeutic options for rare fibrotic diseases are currently available, treatment remains largely supportive, underlining the need for effective treatment. Offering high biocompatibility and the ability to modulate key fibrotic signaling pathways, Mac-EVs demonstrate a promising therapeutic potential. However, significant challenges remain before Mac-EV-based approaches can be effectively implemented in clinical practice.

Nevertheless, considering the excessive immune response observed in rare fibrotic diseases, directly targeting a key immunomodulatory component with Mac-EVs could serve as a preliminary step toward addressing the unmet need for effective treatment. Thus, the strategic modulation of Mac-EVs represents an innovative avenue toward precision therapies for corneal fibrosis in rare diseases, provided that standardization, safety, and functional specificity are rigorously addressed.

## 7. Future Directions

Macrophage plasticity leads to highly variable and context-dependent EV compositions that may deliver unwanted pro-inflammatory cargo. Implementing cell cycle monitoring strategies using cell cycle inhibitors or standardized flow cytometry-based assessment of DNA content and proliferation markers (e.g., Ki-67) could significantly reduce this risk and overall improve Mac-EV quality control. Technical inconsistencies in Mac-EV enrichment, purification, and characterization continue to hamper comparability across studies, underlining the importance of standardized protocols. Furthermore, long-term studies to evaluate the safety and efficacy profile of Mac-EVs in humans are warranted.

## Figures and Tables

**Table 1:** Current treatments and advanced delivery platforms for corneal rare diseases.

Category	Specific Methods/Materials	Main Functions	Limitations or Considerations	Reference
**Supportive treatments**	Preservative-free artificial tears, gels, ointments	Reduce mechanical friction; improve tear film stability	Short duration; require frequent application	[116]
	Antibiotic eye drops	Reduce infection risk after epithelial defects	Risk of antibiotic resistance; need regular drug rotation	[117,118]
	Topical corticosteroids	Anti-inflammatory; delay fibrosis progression	Require IOP monitoring; not suitable for long-term use	[119]
	Non-steroidal anti-inflammatory drugs (NSAIDs)	Pain control; mild inflammation relief	Limited anti-inflammatory effect; possible corneal irritation	[99,120]
	Amniotic membrane extract eye drops	Anti-inflammatory; promote epithelial repair; symptom improvement reported in JEB	Contain growth factors (EGF/FGF/TGF-β)	[100,101]
**Advanced formulations and delivery platforms**	Hydrogels	High adhesion and water content; form a protective layer and provide drug sustained release	Batch variability (especially natural polysaccharides); crosslinkers may irritate ocular surface; tear film may affect gel network stability	[108]
	Nanoparticles	Tunable structure; improve drug solubility and penetration; allow co-loading of multiple agents	Limited effective retention due to blinking and tear turnover; limited drug-loading capacity	[110]
	Liposomes/Cationic nanostructured lipid carriers (CNLC)	Strong adhesion to ocular surface; increase local drug concentration; prolong retention time	Cationic charge enhances adhesion and penetration, but high surface charge may cause epithelial irritation and cytotoxicity	[111]
	Nanogels	Combine advantages of hydrogels and nanoparticles; allow multi-drug loading; can respond to ROS or pH for “on-demand” release	Complex synthesis systems; batch-to-batch consistency difficult to ensure	[113]

**Table 2:** Key biomolecules from EVs for corneal fibrosis.

Category	EV Source	Model	Key Molecules/Pathway	Reference
**MSC-EV**	Induced pluripotent stem cell-derived mesenchymal stromal cells	In vitro: human corneal epithelial cells (hCEpiCs); In vivo: rat anterior stromal injury	miR-432-5p ↓ TRAM2 → ↓ Collagen I/V → ↓ ECM deposition	[139]
	Bone marrow-derived mesenchymal stromal cell	In vitro: hCEpiCs; Ex vivo: human cornea	Not specified	[140]
**CSSC-EV**	Human corneal stromal stem cell	In vivo: mouse stromal injury model; In vitro: human keratocytes	EV miRNA is required for anti-fibrotic function	[115]
	Human corneal stromal stem cell	In vivo: mouse corneal injury model; In vitro: keratocyte	miR-29a, miR-381 ↓ inflammation (iNOS, MCP1, CXCL10) &TGF-β1-induced fibrosis (Col3A1, SPARC, MCP1, FN-EDA, αSMA)	[136]
**Mac-EV**	Rat Peritoneal macrophage	In vivo: rat peritoneal fibrosis; In vitro: human mesothelial cell	miR-204-5p ↓ FOXC1→ ↓ ECM deposition	[141]
	M2-macrophage	In vitro: hCEnCs	↓inflammation (IL-6/IL-1β/ICAM-1)	[142]

**Table 3:** Potential delivery methods of EV-based therapies for general corneal fibrosis.

Delivery Method	EV Cargo	Producing Cell	Model	Results	Year	Reference
Topical eye drops	Not specified	Human corneal mesenchymal stromal cells	Corneal epithelial debridement wound (mouse)	Accelerated epithelial wound closure	2018	[145]
	Not specified	Human bone marrow-derived mesenchymal stromal/stem cells	Alkali burn-induced corneal injury (mouse)	Enhanced corneal wound repair	2022	[150]
	Not specified	Canine mesenchymal stem cells	Stromal ulcer–induced corneal fibrosis (rabbit)	Improved epithelialization and reduced stromal fibrosis	2022	[148]
Hydrogel-based delivery	Not specified	Human corneal stromal stem cells	Stromal injury-induced fibrosis (mouse)	Markedly reduced corneal stromal scarring and fibrosis	2022	[115]
	miR-24-3p	Rabbit adipose-derived mesenchymal stem cells	Alkali burn-induced corneal injury (rabbit)	Accelerated corneal epithelial defect healing and reduced stromal fibrosis	2023	[151]
	Not specified	Mesenchymal stem cells	Corneal defect injury (rabbit)	Rapid epithelialization and reduced fibrosis and inflammatory response	2025	[152]
Subconjunctival injection	miR-21	Human umbilical cord mesenchymal stem cells	Corneal epithelial injury (mouse)	Enhanced corneal epithelial wounds recovery	2022	[153]
	miR-24-3p	Rabbit adipose-derived mesenchymal stem cells	Corneal epithelial defect injury (rabbit)	Accelerated corneal epithelial wound recovery	2023	[151]
Combined (topical eye drops + subconjunctival injection)	miR-27a-3p, miR-27b-3p	Human amniotic epithelial cells	Alkali burn-induced corneal injury (rabbit)	Accelerated corneal epithelial wound healing	2022	[154]

## Data Availability

No new data were created or analyzed in this study. Data sharing is not applicable to this review article.
